# Holographic Gratings for Slow-Neutron Optics

**DOI:** 10.3390/ma5122788

**Published:** 2012-12-12

**Authors:** Juergen Klepp, Christian Pruner, Yasuo Tomita, Peter Geltenbort, Irena Drevenšek-Olenik, Saso Gyergyek, Joachim Kohlbrecher, Martin Fally

**Affiliations:** 1 Faculty of Physics, University of Vienna, Boltzmanngasse 5, 1090 Vienna, Austria; E-Mail: martin.fally@univie.ac.at; 2 Department of Materials Science and Physics, University of Salzburg, 5020 Salzburg, Austria; E-Mail: christian.pruner@sbg.ac.at; 3 Department of Engineering Science, University of Electro-Communications, 1-5-1 Chofugaoka, Chofu, Tokyo 182, Japan; E-Mail: ytomita@ee.uec.ac.jp; 4 Institut Laue Langevin, Boîte Postale 156, 38042 Grenoble Cedex 9, France; E-Mail: geltenbort@ill.fr; 5 Faculty of Mathematics and Physics, University of Ljubljana, Ljubljana 1000, Slovenia; E-Mail: irena.drevensek@ijs.si; 6 The Center of Excellence for Polymer Materials and Technologies, Ljubljana 1000, Slovenia; 7 Materials Synthesis Department, Jožef Stefan Institute, Ljubljana 1001, Slovenia; E-Mail: saso.gyergyek@ijs.si; 8 Laboratory for Neutron Scattering, Paul Scherrer Institute, Villigen PSI 5232, Switzerland; E-Mail: joachim.kohlbrecher@psi.ch

**Keywords:** holographic gratings, neutron diffraction, neutron optics

## Abstract

Recent progress in the development of holographic gratings for neutron-optics applications is reviewed. We summarize the properties of gratings recorded in deuterated (poly)methylmethacrylate, holographic polymer-dispersed liquid crystals and nanoparticle-polymer composites revealed by diffraction experiments with slow neutrons. Existing and anticipated neutron-optical instrumentations based on holographic gratings are discussed.

## 1. Introduction

Neutron-optics experiments and neutron spectroscopy are key techniques for condensed-matter physics [1,2] as well as fundamental physics [3,4]. In view of the number of existing neutron-research centers and the effort put into forthcoming facilities (see, for instance, [5]), further development of neutron-optical techniques in addition to well-established methods is advisable. To advance such development, we intend to use holographic gratings as efficient neutron-optical elements. The purpose of the present review is to summarize recent progress in this field and discuss potential applications as well as forthcoming experiments.

Neutron optics is theoretically based upon the one-particle Schrödinger equation [6], which contains the neutron-optical potential or, equivalently, the neutron refractive-index at a certain wavelength *λ*. The refractive index of a (nonmagnetic) material can be written as

(1) $$\begin{array}{c} n_{0}=\sqrt{1-\frac{V_{N}}{E_{0}}}\approx 1-\frac{\lambda^{2}\;b_{c}\rho }{2\pi }, \end{array}$$

in which $b_{c}$ is the coherent scattering length for a particular isotope; *ρ* is the atomic number density of the material; $V_{N}$ is the optical potential arising from the nuclear interaction; and $E_{0}$ is the energy of the incident neutrons. Note that, in our experiments, $V_{N}/E_{0}$ is several orders of magnitude smaller than 1. If one can tune the value of the refractive index for neutrons as a function of the spatial coordinates, diffractive neutron-optical elements become feasible. This can be done by light-optical holography, for instance. A one-dimensional sinusoidal grating for neutron optics is characterized by the periodically modulated refractive index $n\left(x\right)=n_{0}+\Delta n\cos\left(2\pi x/\Lambda \right)$, with the modulation amplitude

(2) $$\begin{array}{c} \Delta n=\lambda^{2}\;b_{c}\Delta \rho /\left(2\pi \right) \end{array}$$

and grating spacing Λ. The quantity Δρ is the number-density modulation amplitude. In the materials described in the present paper, it is Δρ or—choosing $b_{c}$ via the ingredients of the recording materials—the coherent scattering length density modulation-amplitude $b_{c}\Delta \rho$ that can be set by holography. Note that $b_{c}$ is a real number, *i.e.*, in our theoretical description we assume that incoherent scattering and absorption are negligible. Applications for holographic absorption gratings will be discussed in Section 6.5. Depending on their angle/wavelength selectivity and diffraction efficiency *η*, transmission gratings for neutrons can, in principle, be used for various purposes such as monochromators, wavelength-calibration standards, crystal-collimators, polarizers and mirrors or beam-splitters for matter-wave interferometry with slow neutrons. Mach-Zehnder-type perfect-crystal neutron interferometers have played an important role in investigations of fundamental physics with thermal neutrons [3,7,8,9,10,11,12,13]. For slow neutrons (≲ 5 meV), the Mach-Zehnder geometry was also implemented using artificial structures, such as Ni gratings in reflection geometry combined with mirrors [14], or transmission phase gratings—sputter-etched in quartz glass [15,16]. Furthermore, multilayer-mirrors have also been employed [17,18]. Phase and absorption gratings fabricated by photolithography have been used for neutron phase-imaging and tomography [19,20,21] and small-angle neutron scattering (SANS) [22]. Very-cold neutron SANS (with neutron energies ≲ 0.1 meV), for which holographic gratings could prove useful, is already in the development stage [23].

Artificial grating structures can be produced by exploiting the light-induced change of the refractive index for light—the photorefractive effect. Photorefractive phenomena have been studied intensively in various materials since its discovery in electro-optic crystals [24]. It was noticed only in 1990 [25] that photorefractive (poly)methylmethacrylate can be used in neutron diffraction too. The light-induced change of the refractive index for neutrons was termed the photo-neutron-refractive effect [26]. Using an optical holography setup, signal and reference light beams are superposed at the position of a recording material. If the superposition results in a sinusoidal light intensity pattern—modulating *ρ* for a particular $b_{c}$ due to an intensity-dependent photopolymerization process, say—neutron diffraction gratings are recorded.

The article is organized as follows. In Section 2, some basic explanations of sample preparation and neutron diffraction are given. Section 3, Section 4 and Section 5 contain summaries of the last decade’s results of neutron experiments with gratings recorded in nanoparticle-polymer composites, deuterated (poly)methylmethacrylate and holographic polymer-dispersed liquid crystals. Section 6 describes ideas for future neutron-experiments and potential applications using holographic gratings. We offer a short conclusion and outlook in Section 7.

## 2. Prerequisites

### 2.1. Hologram Recording

**Figure 1:**
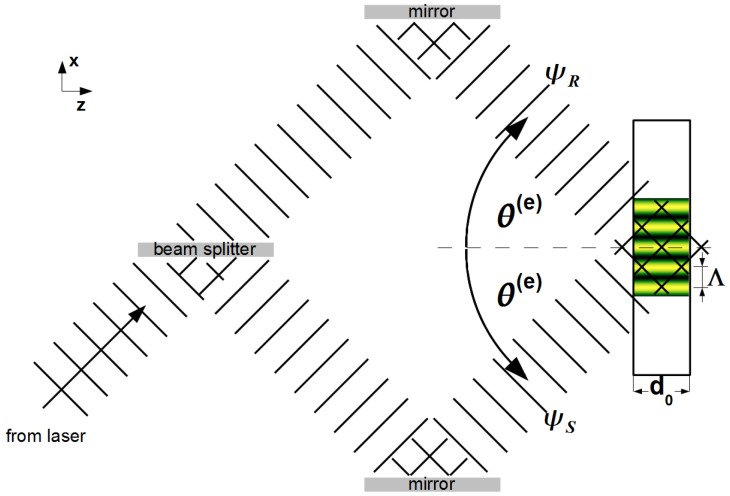
Sketch of the optical holography setup used to record diffraction gratings.

The experimental setup for recording holographic transmission gratings in photosensitive materials is sketched in Figure 1. Two coherent plane s-polarized light-waves, each of amplitude $A_{0}/2$ and wavelength $\lambda_{L}=2\pi /k_{L}$ in free space, are superposed to interfere within the recording material. The *y*-components of their electric fields can be written as:

$$\begin{array}{ccc} \psi_{S} & = & \frac{A_{0}}{2}\exp[ik_{L}(z\cos\theta^{\left(e\right)}+x\sin\theta^{\left(e\right)})],\\ \psi_{R} & = & \frac{A_{0}}{2}\exp[ik_{L}(z\cos\theta^{\left(e\right)}-x\sin\theta^{\left(e\right)})]. \end{array}$$

Each beam encloses the (external) incidence angle $\theta^{\left(e\right)}$ with the sample surface normal. Thus, the resulting light-intensity pattern exhibits a sinusoidal modulation:

$$\begin{array}{c} I=|\psi_{S}+\psi_{R}{|}^{2}=\frac{A_{0}^{2}}{2}\left[1+\cos\left(Kx\right)\right], \end{array}$$

where $K=2\pi /\Lambda =2k_{L}\sin\theta^{\left(e\right)}$. For a given material, illumination time and intensity, the refractive-index change for light may be written as $\Delta n_{0,L}+\Delta n_{L}\cos\left(Kx\right)$. The nature of the particular chemical processes responsible for the modulation of $b_{c}\Delta \rho$, depends on the recording material in use. More details will be given in Section 3, Section 4 and Section 5.

### 2.2. Neutron Diffraction

The slow neutrons used for the experiments described in the present paper, with de Broglie wavelengths in the ranges 0.4 nm <λ< 3 nm and 3 nm <λ< 10 nm, are often termed cold neutrons (CN) and very-cold neutrons (VCN), respectively.

**Figure 2:**
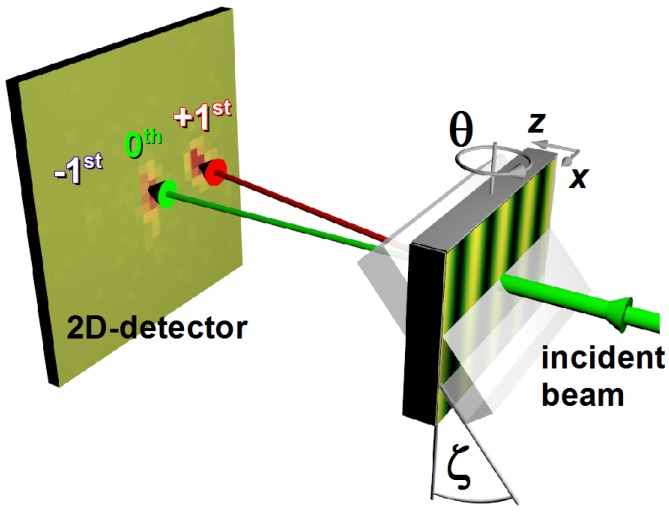
Experimental setup to measure the angular dependence of diffraction (rocking curve) of holographic gratings with neutrons. Upstream, the incident neutron beam is reflected by a supermirror and collimated by a Cd-slit system (both not shown). The sample size typically ranges between 0.8 and 12 cm^2^.

Most neutron diffraction experiments mentioned here were carried out at the instruments SANS I of the SINQ spallation source at the Paul Scherrer Institut (PSI) in Villigen, Switzerland and PF2 of the Institut Laue-Langevin (ILL) in Grenoble, France. The measurement principle is sketched in Figure 2. The gratings are mounted in transmission geometry and tilted to the angle *ζ* around an axis parallel to the grating vector—pointing in *x* direction—in order to adjust the effective thickness (*cf.* Section 3). The incident angle *θ* is varied to measure rocking curves in the vicinity of the Bragg angle $\theta_{B}$ as defined by $\lambda =2\Lambda \sin\theta_{B}$.

Measurements were carried out with various neutron wavelengths. At SANS I of SINQ, the wavelength distribution of the neutrons Δλ/λ, as incident from a velocity selector, is typically 10%. At PF2 of the ILL, VCN with a broad wavelength distribution are available. Here, a Ti/Ni supermirror (multilayer mirror) is necessary to redirect the incident neutron beam to make use of the full collimation length and to obtain a narrower wavelength distribution. The collimation slit widths and distances (SANS I: up to 20 m; PF2: up to 4 m) were mostly chosen so that the typical beam divergence was always better than the expected width of the rocking curve. The latter is approximately given by

(3) $$\begin{array}{c} \mathrm{width}\approx 2\Lambda /d, \end{array}$$

the angular distance between the two minima adjacent left and right to the center, with $d=d_{0}/\cos\zeta$. Here, *ζ* is the tilt angle of the grating (see Figure 2 and Section 3) and $d_{0}$ is the grating thickness for $\zeta =0^{\circ }$. The neutron beam cross-section width and height ranged from 0.5 to 2 mm and 2 mm to 2 cm, respectively, depending also on the size of the sample. At PF2, a setup for measurement of the time of flight (TOF) of neutrons in successive pulses (created by a disk chopper) to the detector was available to study the wavelength dependence of the diffraction efficiency. Depending on available flux, collimation and particular sample, typical measurement times per setting of *θ* range from a couple of minutes to several days for TOF measurements at PF2. In both setups, 2D detectors were used. The spatial resolution of such detectors is typically in the range of some mm. Since the grating period Λ is large compared with the wavelength of the incident neutrons, $\theta_{B}\approx \lambda /\left(2\Lambda \right)$ is of the order of $0.1^{\circ }$. Depending on the spatial resolution of the detector system, a distance of some meters has to be maintained between the grating and the detector to obtain well-separated diffraction spots. For a more complete description of SANS I and PF2 see, e.g., References [27,28], respectively.

### 2.3. Diffraction Efficiency

**Figure 3:**
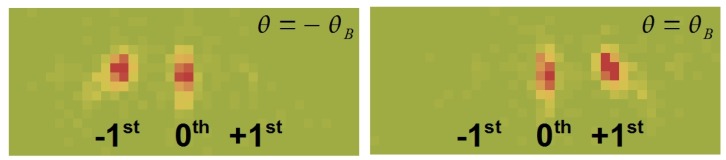
Diffraction pattern of a nanoparticle-polymer composite grating (20 vol% SiO_2_ nanoparticle concentration, Λ=0.5 *μ*m, $d_{0}\approx 108$ *μ*m) set to the Bragg positions. The measurement was done with a ^3^He multi-wire proportional chamber. The position resolution is 2 × 2 mm^2^. Efficiency: 80% at λ=4 nm). Only two diffraction spots are observed. The grating operates in the two-wave coupling regime.

Experimentally, the diffraction efficiency for the *i*th diffraction order is defined as:

(4) $$\begin{array}{c} \eta_{i}=I_{i}/I_{\mathrm{tot}}, \end{array}$$

where $I_{i}$ and $I_{\mathrm{tot}}$ are the measured intensities of the *i*th diffraction order and the total intensity of forward-diffracted (transmitted) and diffracted beams, respectively. For each *θ*, the sum over all 2D-detector pixels in each separated spot (*cf.* Figure 3)—associated to one single diffraction order—is taken and the obtained intensities plugged into Equation (4) to obtain the diffraction efficiency $\eta_{i}$ for the *i*th diffraction order at a certain incident angle *θ*.

It is convenient to adopt Kogelnik’s two-wave coupling theory for Bragg diffraction of light by holographic volume phase gratings [29] to write the diffraction efficiency for neutrons in the symmetric Laue-case (transmission geometry) as:

(5) $$\begin{array}{c} \eta =\nu^{2}\frac{\sin^{2}\sqrt{\nu^{2}+\xi^{2}}}{\nu^{2}+\xi^{2}}, \end{array}$$

with

(6) $$\begin{array}{c} \nu =\frac{\lambda \;d\;b_{c}\Delta \rho }{2\cos\theta }\;\;\mathrm{and}\;\;\xi =\frac{\pi d(\theta_{B}-\theta )}{\Lambda }. \end{array}$$

**Figure 4:**
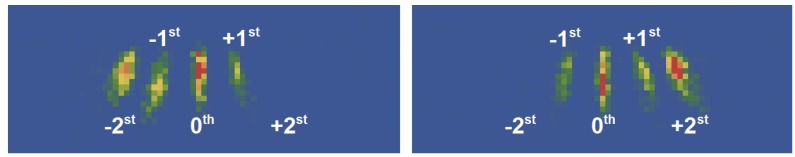
Diffraction pattern at two different incident angles *θ*. Grating parameters: Λ=1 *μ*m, $d_{0}\approx 185$ *μ*m, 20 vol% SiO_2_ nanoparticles. The used wavelength was λ≈3.8 nm. Also 2nd order diffraction spots are clearly visible here at these single *θ*-values, which means that the assumption of two-wave coupling—a prerequisite for Equation 5—is not justified.

Depending on the parameters of a purely sinusoidal grating, more than two diffraction spots can show up at a certain *θ* (see also Figure 4) and diffraction does not occur in two-wave-coupling regime. On the other hand, if the modulation of Δn is not purely sinusoidal, the assumption of two-wave coupling can still be reasonable as long as the grating is thick enough. Terminology and technical details regarding diffraction regimes are discussed in Reference [30]. Note that Equation (5) is equivalent to the expression for the reflectivity of thick crystals in transmission geometry derived from dynamical diffraction theory [6], as was discussed in Reference [31].

Mainly, three material classes have been investigated for holographic production of diffractive elements for neutrons. In the following sections, the properties, advantages and drawbacks of those are reviewed.

## 3. Nanoparticle-Polymer Composite gratings

It is only a few years ago that nanoparticle-polymer composites were considered for producing neutron-optical elements [32]. Inorganic nanoparticles (NPs) embedded in a photopolymer matrix had already been investigated intensively [33,34,35,36,37,38,39] for light-optics applications.

One great advantage of NP-polymer composites—which has not yet been exploited in the experiments done so far—is that the refractive-index modulation can be tuned by choosing the species of NPs. One can select a particular species with a scattering length appropriate for a specific neutron-optics application. Like that, mirrors and beam splitters (*cf.* Section 6.3), absorption gratings (*cf.* Section 6.5) and polarizers (*cf.* Section 6.4) become feasible. Also, including NPs in the polymer matrix increases the mechanical stability, *i.e.*, shrinkage—typical for polymerisation processes also in deuterated (poly)methylmethacrylate—is strongly reduced [33,40].

The NPs used for neutron diffraction studies (mostly SiO_2_ NPs) have an average core diameter of about 13 nm with size distribution of approximately ±5%. They are produced by liquid-phase synthesis and dissolved in a methyl isobutyl ketone solution. The NP sol is dispersed to (meth)acrylate monomer. A photoinitiator is added to enable the monomer to polymerize on illumination with light. The mixture is cast on a glass plate and is dried. Spacers are arranged around the sample before it is covered with another glass plate to obtain film samples ready for being structured by a spatial light-intensity pattern. Before recording, the photoinitiator, the monomer and the NPs are homogeneously distributed in the sample material. Via the photoinitiator the light-intensity pattern induces polymerization in the bright sample regions, a process that consumes monomers that diffuse from dark to bright regions [35]. As a consequence of the growing monomer-concentration gradient, NPs move from bright to dark regions, resulting in an approximately sinusoidal NP-concentration pattern, which can be used as neutron diffraction grating. Subsequent homogeneous illumination ensures that the material is fully polymerized so that the NP density-modulation remains stable for at least some years. Stability has been confirmed by constantly checking the properties of certain samples with light for nearly ten years. Further details on the sample preparation technique can be found in the references mentioned above.

**Figure 5:**
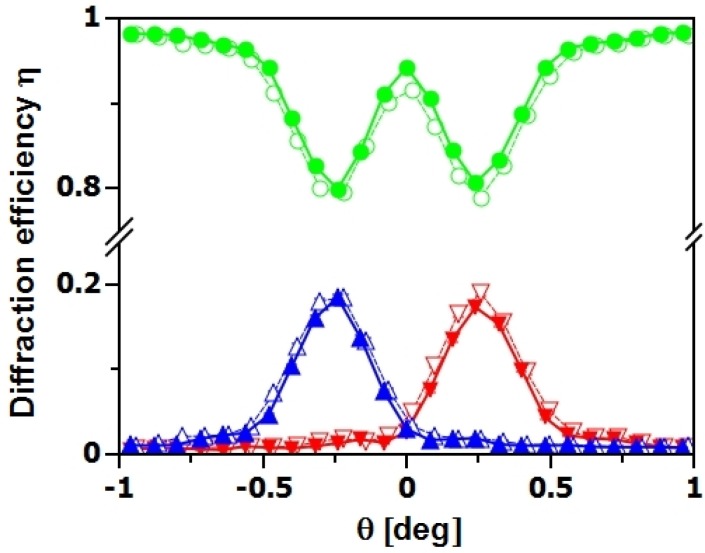
Rocking curves of NP-polymer composite gratings with 20 vol% (▴⋯-1st order, ●⋯0th order, ▾⋯+1st order) and 25 vol% (▵⋯-1st order, ○⋯0th order, ▿⋯+1st order) SiO_2_ NP concentration (λ≈4.5 nm, Λ=0.5 *μ*m, $d_{0}\;\approx \;80$ *μ*m). Curves were measured using exactly the same experimental parameters. The neutron diffraction-efficiency does not seem to be very sensitive to changes of the NP concentration in this range.

It was shown that plane-wave gratings of up to $d_{0}=100$ *μ*m can be produced. However, at around 100 *μ*m thickness, holographic light scattering [41,42,43], that smears out the recording light-intensity pattern within thick NP-polymer gratings [44], becomes apparent. As a result, Δn decays exponentially along the sample depth—the distance as measured from the sample front surface in the direction of the grating thickness. Such a behavior was described by Uchida [45]. From a fit of the measured rocking curve to Uchida’s model, one can extract the sample depth after which Δn has reduced to 1/e of its value at the front surface and $\langle b_{c}\Delta \rho \rangle$—the coherent scattering length density modulation amplitude as averaged over the sample thickness. Typical values of $\langle b_{c}\Delta \rho \rangle$ obtained in this way are around 3 *μ*m^-2^ [46], which corresponds to $\Delta n\approx 7.6\times 10^{-6}$ for λ=4 nm. In the latter reference, improvement of NP-polymer composite gratings by variation of sample parameters has been assessed. After detailed studies with light [34] and preliminary results of neutron experiments, a pronounced maximum of Δn as a function of the NP concentration was hoped for in the region around 25 vol% (corresponding to $\rho \approx 2.2\times 10^{23}m^{-3}$), but could not be confirmed (see Figure 5). Obviously, the two curves for 20 vol% and 25 vol% NP concentrations are not much different and for the moment it seems that the neutron diffraction efficiency is surprisingly insensitive to this parameter. However, there are also results that indicate improvement at 25 vol% (see Table 1, S#10 (2011b) and S#11 (2011b)). This issue needs further investigation. It was shown in Reference [46] that a short grating period is unfavorable for high diffraction efficiency as it seems to be the case for many acrylates [32,34,39]. This means that for all applications in which the magnitude of the Bragg angle is not of major importance, long grating periods should be preferred to achieve higher diffraction efficiency. However, it was also noted in [46] that larger Λ seems not to prevent decay of Δn for larger thickness.

A work-around to the limitation in thickness is to use the interference effects occurring in diffraction by thick periodic structures: Sippel [47] and, a few years later, Shull [48] demonstrated the Pendellösung interference effect for neutrons that is predicted by dynamical diffraction theory [3,6,49,50] and also by its fellow theories developed for visible light [29,45,51]. Apart from neutrons, the effect has been observed for X-rays [52], *γ*-rays [53], infrared [54], visible light (see, for instance, [55,56]) and atoms diffracted by standing light-waves [57]. Upon entering a crystal or a holographic grating at the Bragg condition, the wavevector component of the neutron beam parallel to the surface is conserved, since phase matching at the boundary is required. However, the component normal to the boundary is split in two, resulting in two standing waves inside the material—one in phase and the other out of phase with the periodic potential. The interference of these waves results in an oscillation of the neutron flux at the exit surface between diffracted and forward-diffracted beams depending on the grating thickness [47], the incident wavelength [48] and the coherent scattering length density modulation amplitude. The relevance of the Pendellösung effect for neutron diffraction in holographic gratings has been pointed out in Reference [31]. There, based on Kogelnik’s theory, the expression for the oscillation of the first order diffraction efficiency at the Bragg peak was written as:

(7) $$\begin{array}{c} \eta_{P}=\sin^{2}\left(\frac{\pi d}{\Delta }\right). \end{array}$$

**Figure 6:**
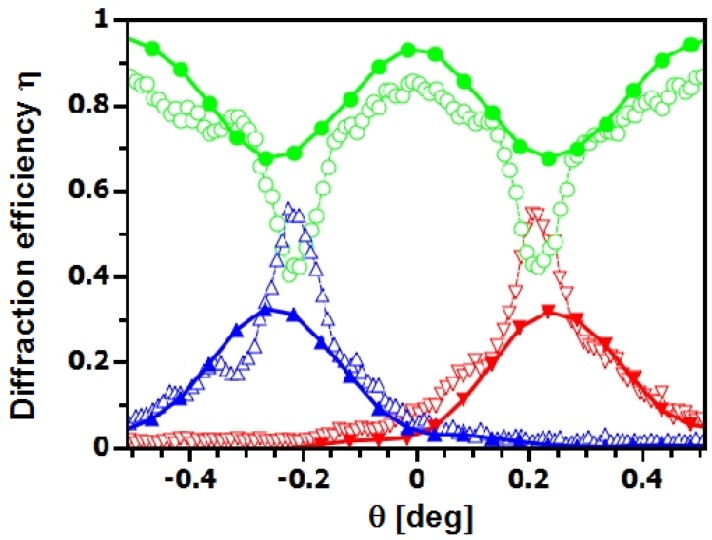
Rocking curves measured at λ≈4.4 nm for zero tilt (▴⋯-1st order, ●⋯0th order, ▾⋯+1st order) and $\zeta =68^{\circ }$ (▵⋯-1st order, ○⋯0th order, ▿⋯+1st order). Grating parameters: Λ=0.5 *μ*m, $d_{0}\approx 115$ *μ*m, 20 vol% SiO_2_ NPs. The slight shift of the tilted peaks toward θ=0 and their unexpectedly low $\eta_{P}$ is due to the broad incident wavelength distribution, as simulations indicate.

Here, Δ is usually called Pendellösung period or extinction length and is given by:

(8) $$\begin{array}{c} \Delta =\frac{2\pi \cos\theta_{B}}{\lambda \;b_{c}\Delta \rho } \end{array}$$

for holographic gratings. Δ is in the range of 1 mm for holographic NP-polymer gratings at slow-neutron wavelengths. The Pendellösung effect has also been observed by tilting crystals around an axis parallel to the grating vector, so that essentially its effective thickness *d*, appearing in Equation (7), is increased [58]. Whenever increasing the recorded grating thickness is impossible because of unwanted light-scattering in the recording process, one can increase the effective thickness of a thin grating by tilting. An example is shown in Figure 6.

**Figure 7:**
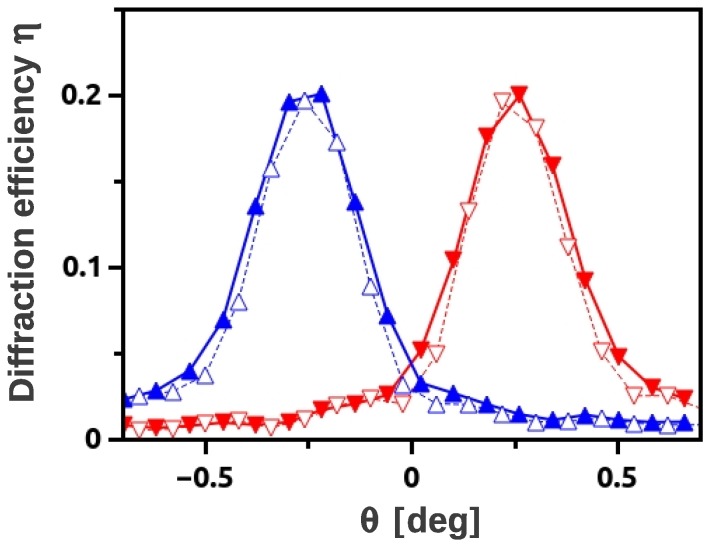
Rocking curve measured with λ≈4.5 nm before (▴⋯-1st order, ▾⋯+1st order) and after (▵⋯-1st order, ▿⋯+1st order) removal of the covering glass plates, without changing the experimental setup between the measurements. Grating parameters: Λ=0.5 *μ*m, $d_{0}\approx 92$ *μ*m, 20 vol% SiO_2_ NPs.

**Figure 8:**
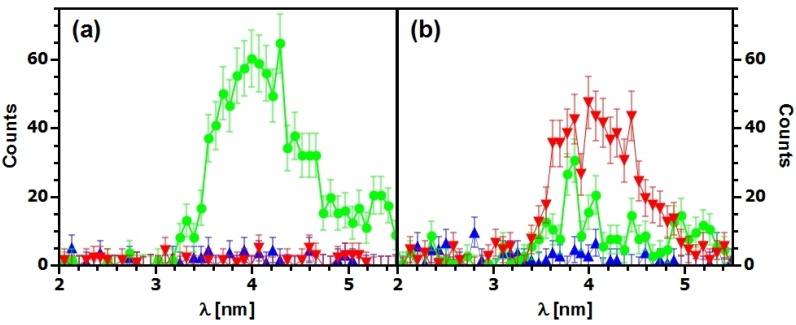
Background-corrected wavelength spectra of the intensity diffracted by a SiO_2_ NP-polymer composite grating at tilt angle $\zeta \approx 70^{\circ }$ for (a) *θ* far from $\theta_{B}$; and (b) $\theta =\theta_{B}$ (▴⋯-1st order, ●⋯0th order, ▾⋯+1st order). The measurement time at each *θ* is several days mainly due to the chopper disc that has an open-to-closed ratio of about 3%. The distance between the collimation slits (size: $1\times 4{\mathrm{mm}}^{2}$ and $0.5\times 2{\mathrm{mm}}^{2}$) is about 2.1 m.

The feasibility of a beam splitter for CN has been demonstrated by such means [46,59]. The remaining limitations of neutron-optical elements based on NP-polymer composites have been the small grating diameter—decreasing the possible beam size upon tilting—and incoherent scattering/absorption of sample glass covers and the sample itself. Consequently, neutron diffraction experiments with free-standing NP-polymer film-gratings have also been carried out. Here, the glass plates used for preparation and recording carry a silane layer so that the plates can be removed easily from the polymer sample-surface after recording. In Figure 7, rocking curves for a grating before and after removal of the glass plates are shown. Clearly, the optical quality of the grating is not deteriorated by removal of the glass plates. Additionally, the resulting thin film-gratings were recorded with a beam of about 2 cm diameter. This large diameter—together with a perfectly homogeneous grating structure across the entire sample area—makes it possible to go to large tilt angles and still use an incident neutron beam of considerable height of a couple of millimeters. With such a free-standing film grating with Λ=0.5 *μ*m, $d_{0}\approx 108$ *μ*m and 20 vol% SiO_2_ NP concentration, 90% diffraction efficiency, *i.e.*, mirror-like behavior, was achieved for a neutron wavelength of about 4.1 nm [60]. The necessary wavelength resolution to observe such high diffraction efficiency was achieved by using a TOF system. Typical TOF spectra are shown in Figure 8.

The next step to improve NP-polymer composites for neutron optics is to use deuterated monomer for preparation to avoid the bigger part of loss (wavelength dependent; roughly 20% for λ=4 nm) due to incoherent scattering and absorption by hydrogen. Also, the main advantage of such materials—the possibility to select $b_{c}$ by choosing the NP species—is yet to be investigated and is expected to yield even better values for $b_{c}\Delta \rho$ for new applications (see Section 6). For instance, diamond NPs—which are already being used for slow-neutron reflection [61]—could be ideal for holographic neutron phase-gratings because of their high coherent scattering length and their low absorption cross-section.

## 4. (Poly)methylmethacrylate Gratings

Since it was noticed that holographic gratings recorded in (poly)methylmethacrylate (PMMA) exhibit diffraction of CN [25,62], the method has undergone several major steps of development: Using a different photoinitiator along with substitution of hydrogen—which shows strong incoherent scattering and absorption of neutrons—contained in the recording material by deuterium and increase of the grating diameter [63] are of importance. Also, it was noted that nonlinear processes during photo-polymerization of d-MMA lead to higher harmonics with Λ/2,Λ/3⋯ that—depending on Λ—can often not be detected by light-optical means [64] even if *produced* by optical holography. A substantial decrease of the grating period and, therefore, larger Bragg angles are reached by controlled enhancement of higher harmonics.

In short, the actual method for preparation of the photosensitive d-PMMA samples is as follows: After separation of liquid d-MMA monomer from a polymerization inhibitor, a thermal initiator and a photoinitiator are added. The degree of substitution of hydrogen by deuterium is 99.7%. The mixture is injected into cuvettes that consist of quartz glass front- and back-windows. Constant spacing of a few millimeters between the windows is provided by O-rings. To compensate volume shrinkage during polymerization, moderate stress is applied to the windows by metal springs. Thermal pre-polymerization of the liquid d-MMA mixture is done for 48h at a temperature of 45° to reach about 80% of polymerization. The remaining 20% are available for light-induced polymerization, *i.e.*, hologram recording as described in Section 2.1.

**Figure 9:**
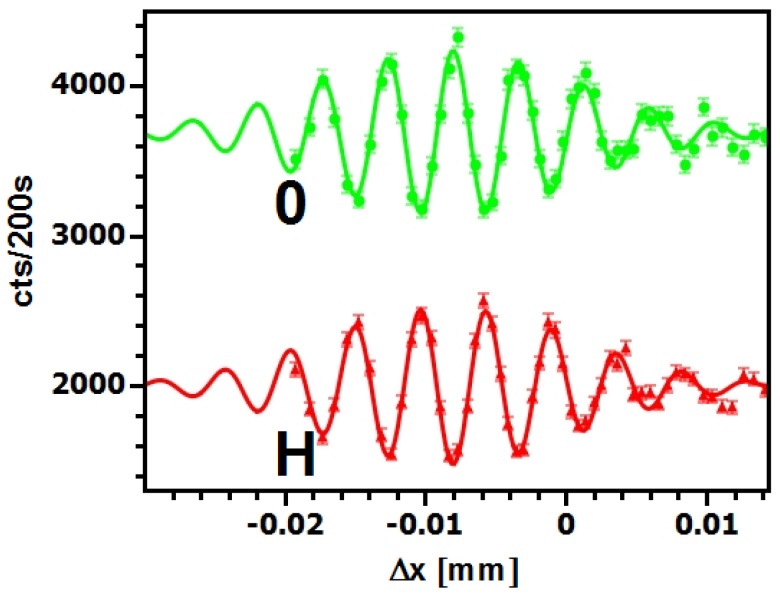
Intensity oscillations measured at the instrument D22 of the ILL with the d-PMMA grating-interferometer for CN [66]. Δx is the path-length difference between left and right interferometer path induced by stepwise rotation of a sapphire phase-shifter slab in the interferometer.

The efforts concerning d-PMMA culminated in successful tests of the first Mach-Zehnder neutron interferometer based on holographic gratings [65] with grating period Λ=798 nm for a neutron wavelength λ=1.5 nm. The maximum visibility was only about 15%, which is mainly due to the small beam separation: Neutrons that are forward diffracted at the second grating of the interferometer cannot be blocked and increase the background of the signal measured by the detector. This problem can be solved either by recording gratings with smaller period, that result in larger Bragg angles, or using mirror gratings with η≈1, as achieved recently with NP-polymer composite gratings (see Section 3). For the d-PMMA interferometer, accurate adjustment of the three gratings with respect to each other was achieved by fixing three cuvettes—containing the recording material—on a rigid chassis. The three holograms are then recorded by moving the chassis such that each cuvette is positioned at the crossing of the coherent laser beams successively. For positioning, the chassis is mounted on a translation stage. Using a polarization-optical setup for piezo-driven roll compensation and autocollimator-controlled compensation for pitch and yaw of the translation stage, it could be assured that the misalignment of the grating vectors was less than about 1 *μ*rad [66]. Furthermore, in the latter reference the grating period was decreased to Λ=380 nm and the total length of the interferometer was increased by a factor of 5. Using a neutron wavelength of λ≈2.6 nm, the beam separation at the second grating was about 1mm. The maximum visibility of the interference fringes measured with this interferometer was 26%, as is shown in Figure 9.

Diffraction gratings recorded in d-PMMA exhibit high diffraction efficiency, but the recorded patterns are—due to continuing polymerization processes—not stable and change their diffraction properties. The grating structure deteriorates when exposed to light or heat. While such gratings are still very useful for fundamental physics experiments, stability on a time scale of at least some years is highly desirable for neutron-optical elements employed in neutron scattering instruments for day-to-day use. An overview of neutron optics with d-PMMA gratings is given in Reference [26].

**Figure 10:**
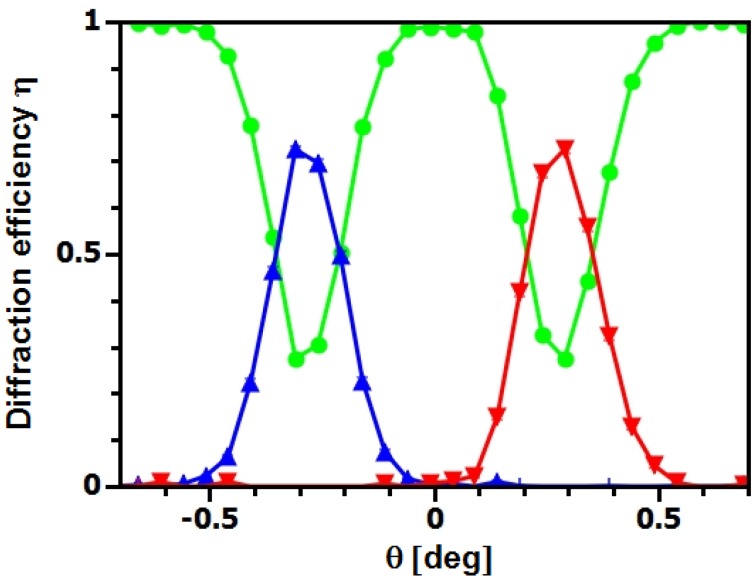
Rocking curve of a d-PMMA grating with Λ=381 nm measured at λ≈3.8 nm (▴⋯-1st order, ●⋯0th order, ▾⋯+1st order). The value of $b_{c}\Delta \rho$ is unknown (see text). The thickness of the sample material, as given by the distance of the quartz glass plates, is 3 mm.

**Figure 11:**
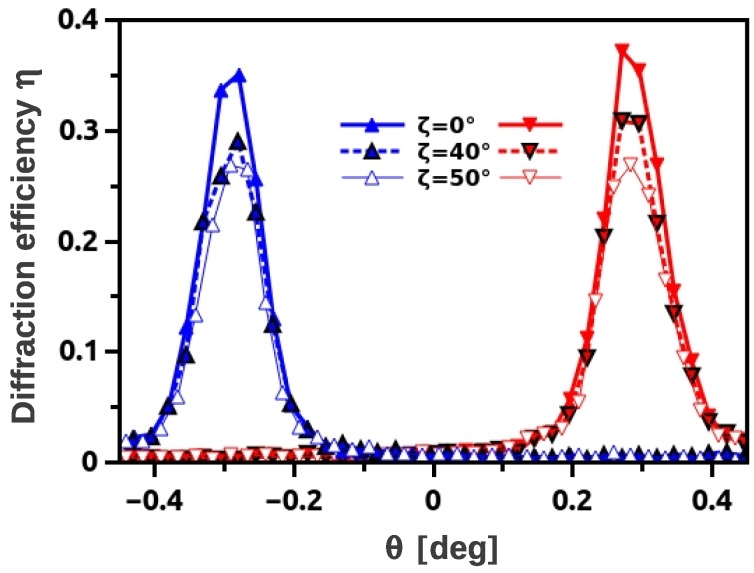
Rocking curves of a d-PMMA grating (Λ=381 nm) measured at λ≈3.8 nm and different tilt angles *ζ*.

Plotting Equation (5) for typical parameters of single-crystals (see, for instance, [3,6]), one can see that the diffraction efficiency *η* is rapidly oscillating as a function of the deviation $\theta -\theta_{B}$ from the exact Bragg angle. This is mainly due to their large $b_{c}\Delta \rho$ and their thickness of several millimeters. As a consequence, such oscillations are difficult to observe for reasonable values of the neutron-beam collimation. Instead, measured rocking curves display an average over these oscillations. The d-PMMA gratings used in Reference [66] are roughly 2mm thick, as can be estimated from light-optical measurements (not shown). Therefore, it can be assumed that the measured rocking curves also suffer from averaging. This assumption is supported by the fact that the example in Figure 10 does not exhibit clear side maxima in contrast to the curves measured with the much thinner holographic polymer-dispersed liquid-crystal gratings, for instance (see Section 5). Furthermore, using Equation (3) one would expect the rocking curve to have a width of approximately $0.02^{\circ }$, which cannot be observed with the beam divergence set to $0.1^{\circ }$, as in the case of Figure 10. It is difficult to fit Equation (5) to such data in order to obtain an estimation of $b_{c}\Delta \rho$ for d-PMMA gratings. Apart from a large beam divergence, also inhomogeneities of the gratings can be a reason for smeared-out fine structures of rocking curves. In Figure 11, rocking curves measured with a tilted d-PMMA grating (Λ=381 nm) are shown. They are again way too wide and lack observable side maxima. Assuming that πd/Δ≫π/2 in Equation (7), so that averaging occurs because of insufficient beam collimation, a diffraction efficiency below 0.5—as observed—is not expected. Furthermore, tilting results in a decrease of $\eta_{P}$. This indicates that such gratings operate in a regime in which πd/Δ>π/2 (over-modulation or over-coupling), but not excessively large. In this case, averaging and Pendellösung interference are at work in parallel.

## 5. Holographic Polymer-Dispersed Liquid Crystal gratings

Holographic polymer-dispersed liquid crystals (H-PDLC) are intensively investigated candidate materials for holographic optical elements [67,68]. Before recording, they consist of a homogeneous mixture of a photosensitive prepolymer and liquid crystals. Upon illumination with a spatially modulated light-intensity pattern, periodic structures are recorded. The underlying process is a photopolymerization reaction that takes place more rapidly in the bright regions of the light-intensity pattern than in dark regions and consequently the monomers diffuse to the bright regions while the liquid crystalline molecules stay in the dark regions [69]. Additionally, when a certain degree of photopolymerization is reached, demixing of the compounds takes place. Liquid crystal droplets, embedded in a polymer matrix, are formed. That way, optical holograms with high spatial modulation of the refractive index for light are recorded. From a light-optics point of view, such gratings are anisotropic, *i.e.*, the diffraction efficiency depends on the polarization state of the incident laser beam [70]. The orientation of liquid crystals in the droplets and, hence, optical properties can be controlled by an external electric field. This is the major advantage of H-PDLCs over other materials and keeps great potential for light-optical applications.

**Figure 12:**
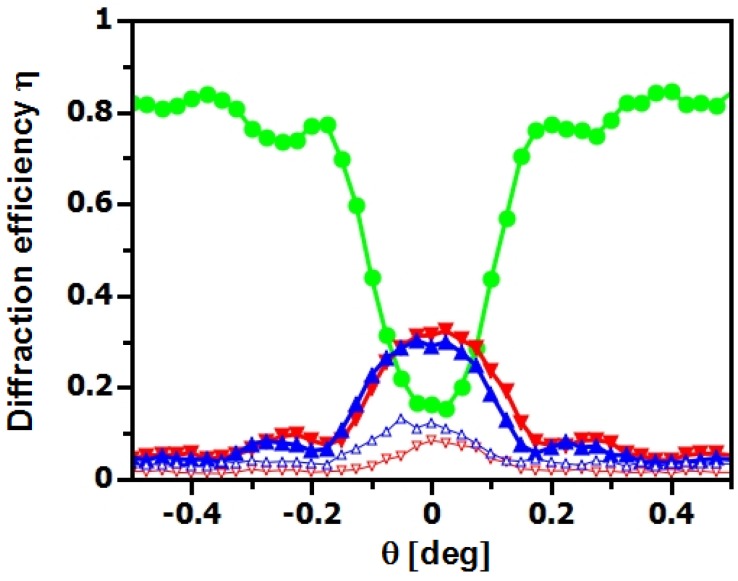
Rocking curve of an H-PDLC grating measured at λ≈4.4 nm (▴⋯-1st order, ●⋯0th order, ▾⋯+1st order, ▵⋯-2nd order, ▿⋯+2nd order). Grating parameters: Λ=1.2 *μ*m, $d_{0}=30$ *μ*m.

A photosensitive mixture is prepared from commercially available components (curable prepolymer, nematic liquid crystal, acrylate). The ratio of constituents is chosen according to literature (see, for instance, Reference [71]). The mixture is placed between two glass plates separated by suitable spacers. While studying the phase separation processes in H-PDLC-gratings with neutron scattering methods, it has also been noted that such gratings exhibited the record of $b_{c}\Delta \rho \approx 10$ *μ*m^-2^ for holographic gratings [72] at that time. The best diffraction efficiency reached so far is about 12% at a neutron wavelength of 2 nm, which holds some potential for neutron optics. An example of a measured rocking curve is shown in Figure 12. The main obstacles are (i) the limited recording thickness that is due to detrimental light scattering during hologram recording [42]; and (ii) that small Λ have negative influence on the diffraction efficiency in H-PDLCs as it is also the case for NP-polymer composites. The former problem can—to a certain extent—be overcome by tilting, as described in Section 3. The latter issue was investigated using neutrons in Reference [32].

## 6. Perspectives and Potential Applications

### 6.1. Pendellösung Oscillation

As explained in Section 3, due to the quantum-mechanical superposition of several neutron waves formed in a periodic potential, the neutron intensity is swapped back and forth between diffracted and forward-diffracted beams as a function of wavelength or effective thickness of the potential region. The latter can be increased by tilting crystals [58] or holographic gratings of limited thickness [46] around an axis parallel to the grating vector. Therefore, this so-called Pendellösung interference effect allows to control the diffraction efficiency to a certain extent.

**Figure 13:**
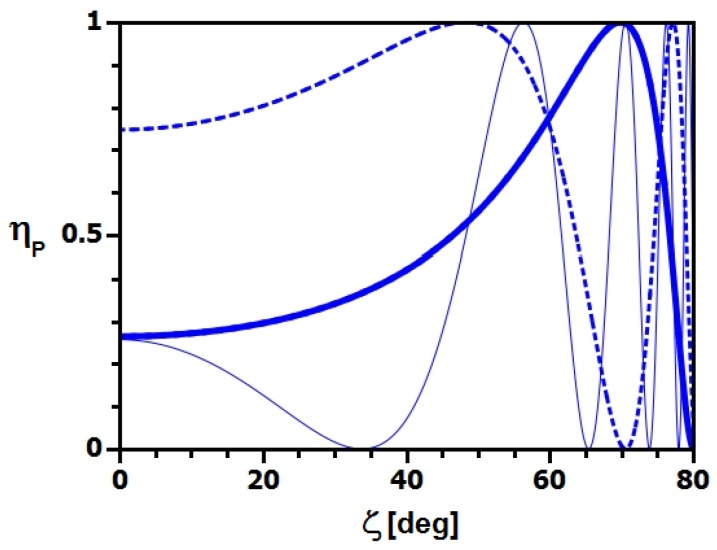
Pendellösung oscillations: $\eta_{P}$ versus *ζ* calculated for the grating parameter sets Λ=0.5 *μ*m, $d_{0}=100$ *μ*m, $b_{c}\Delta \rho =2.7$ *μ*m^-2^ (thick solid line) and Λ=1 *μ*m, $d_{0}=90$ *μ*m, $b_{c}\Delta \rho =5.8$ *μ*m^-2^ (dashed line) at λ=4 nm. For the second parameter set, the oscillation is also shown for λ=10 nm (thin solid line).

In Figure 13, the oscillation of the peak diffraction efficiency $\eta_{P}$ according to Equation (7) is plotted as a function of the tilt angle *ζ* for three parameter sets: First, $\eta_{P}$ is shown for a standard grating with an incident neutron wavelength of 4 nm (thick line). Such gratings were used in References [46,59,60] because they operate in the two-wave coupling regime (Bragg regime) so that no intensity is lost to unnecessary diffraction orders and—at the same time—the gratings exhibit high diffraction efficiency for relatively small grating spacing (large $\theta_{B}$), which is important for applications. The theoretical prediction given by the thick line in Figure 13 was tested in the latter two references given above, but only up to $\zeta \approx 70^{\circ }$. At this angle, the grating acts as a mirror (see also Section 3). To measure a full Pendellösung oscillation with such a grating, it would be necessary, say, to increase *ζ* to slighly more than $80^{\circ }$, for the data to include at least one $\eta_{P}$ that is again larger than the minimum (*cf.* Figure 13). At this tilt angle the effective thickness is about 580 microns and the expected width of the rocking curve is only about 1mrad. It is inefficient to measure at λ≈4 nm with the necessary horizontal collimation. Furthermore, a grating of circular area with radius of about 2 cm appears only 3 mm high and the incident beam height has to be diminished accordingly also in vertical direction. If the value of $\theta_{B}$ is not of importance, the situation can be improved for a variation of the grating parameters as shown by the dashed line of Figure 13. With Λ=1μm at still roughly 100 microns thickness, also $b_{c}\Delta \rho$ increases and the grating exhibits a theoretical diffraction efficiency of about 0.75 already for $\zeta =0^{\circ }$ for λ=4 nm. The first minimum of $\eta_{P}$ would be encountered at *ζ* only slightly larger than $70^{\circ }$, which is reasonable for a proof-of-principle experiment. The above is, of course, only valid as long as Equation 5 is applicable. The conditions for this experiment become even better when longer wavelength are available at considerable flux. For instance, the thin blue line in Figure 13 is the theoretical prediction of the second (above)—more favorable for the purpose—parameter set for λ=10 nm, which is about the longest VCN wavelength used for experiments reported in literature [16]. Here, $\zeta ≲68^{\circ }$ already contains the necessary measurement range.

Because of the rapid oscillations of $\eta_{P}$ for large tilt angles shown on the right of Figure 13, it is also tempting to assess the sensitivity of the setup in Figure 2 to small deviations in *ζ* or, equivalently, deviations in the incident angle of the incoming neutron beam with respect to the tilted sample. For instance, assuming a hypothetical neutron charge one order of magnitude smaller than the current experimental upper limit [73] at about $10^{-41}$ C, neutrons with wavelength λ=10 nm that traverse a static electric field of $\pm 6\times 10^{6}$ V/m and 10 m length would be deflected by an angle as small as $\Delta \zeta =4.6\times 10^{-10}$ rad upon reversal of the field polarity. Such deflection of the beam, incident to the stronger grating used for the simulations for Figure 13 at $\zeta =85^{\circ }$ (effective grating thickness ≈1.1 mm, expected rocking curve width ≈0.002 rad), would result in a change of the peak diffraction efficiency as small as $\Delta \eta_{P}=2\times 10^{-7}$, which is rather difficult to observe. Vice versa, assuming that we can detect $\Delta \eta_{P}=0.001$ in reasonable time, we reach an angular sensitivity of $\Delta \zeta =2\times 10^{-6}$ rad. This value—although respectable—is 4 orders of magnitude from challenging methods using ultra-cold neutrons that are currently being developed [74] for improving the experimental limits of the neutron charge. Still, at this stage of development of holographic gratings, one can observe small vertical deflections as predicted from phase matching conditions at the grating surface that naturally arise from certain assumptions in diffraction theories. Such deflections have recently been studied with light [75] and are still to be observed for matter waves. The expected deviation angles are of the order of $\Delta \zeta \approx 10^{-5}$ rad for λ=10 nm.

### 6.2. Grating Collimators

**Figure 14:**
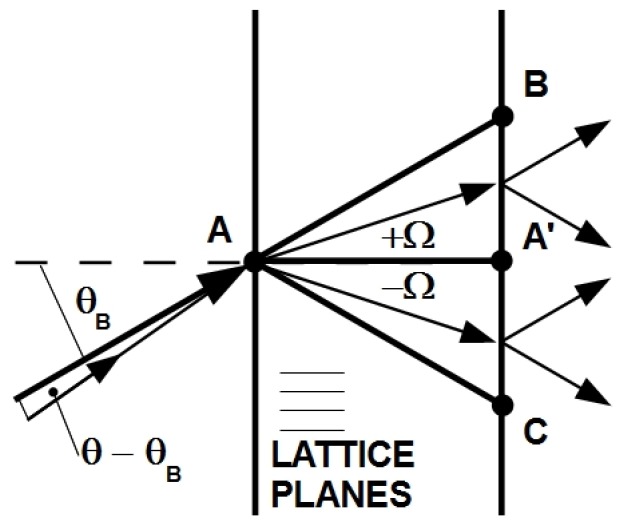
Angle amplification effect. Direction of neutron currents for small deviations from the Bragg position.

We shortly explain the angle-amplification effect in silicon crystals with the help of Figure 14 (see also Reference [3], Chapter 10). In dynamical diffraction theory applied to neutron diffraction by perfect crystals in symmetric Laue-geometry, the directions of diffracted and transmitted neutron currents within the crystal sweep over the entire Borrmann triangle ABC for arcsecond-deviations $\theta -\theta_{B}$ from the exact Bragg condition. This means the transmitted (diffracted) neutron current propagates across the crystal at an angle +Ω (-Ω) with respect to the lattice planes. Ω and the misset angle $\theta -\theta_{B}$ are related by

(9) $$\begin{array}{c} \Omega =\frac{2E_{0}\sin^{2}\theta_{B}}{|V_{H}|}\left(\theta -\theta_{B}\right), \end{array}$$

where $|V_{H}|$ is the strength of the periodic potential for a particular Bragg-reflection (corresponding to $2\pi \hbar^{2}b_{c}\Delta \rho /m$). For silicon crystals the amplification factor can be of the order of $10^{5}$. Note that, for $\theta -\theta_{B}=0$, the neutron currents propagate parallel to the lattice planes, along the line $A-A^{\prime }$. It was suggested to use crystals in such a configuration as collimators/monochromators by selecting only the part of diffracted neutrons that fulfill the Bragg-condition to a desired accuracy [76]: If narrow absorber slits are mounted at the front and the back face of the crystal, we can filter neutrons that deviate either in wavelength, direction or both, *i.e.*, neutrons that do not fulfil the Bragg condition to a given accuracy. This interesting effect has also been used in high wavelength-resolution experiments and proposals (see, for instance, [76,77,78]). Assuming a d-PMMA grating with realistic parameters Λ=200 nm, $d_{0}=5$ mm and $b_{c}\Delta \rho =10\mu m^{-2}$ at λ=10 nm it follows that $\theta_{B}=1.4^{\circ }$ and, therefore, the amplification factor is roughly 24, which might still prove useful for collimation/monochromatisation tasks. However, due to the small Bragg angle, the distance between points $A^{\prime }$ and B/C is only about 250 microns for $\zeta =60^{\circ }$, say. It is therefore difficult to select the useful fraction of the incident intensity without substantial losses. At the present stage of development, holographic gratings are not suitable for this purpose. The grating thickness must at least be doubled and/or their grating spacing halved. Alternatively, several free-standing d-PMMA gratings might be readily used to form stacks that can also be tilted. Note that the angle-amplification effect should also occur for light, for which it has perhaps not been investigated yet.

### 6.3. Mach-Zehnder Interferometer

**Figure 15:**
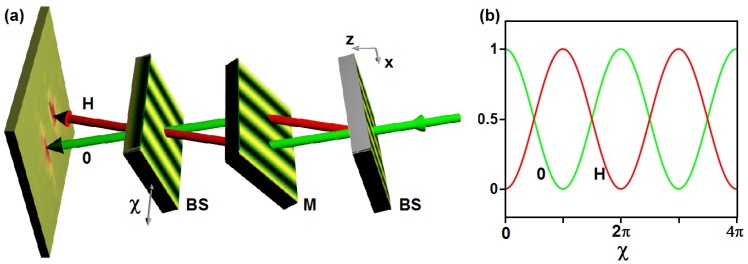
(**a**) Mach-Zehnder interferometer of the triple-Laue type using three NP-polymer composite gratings; (**b**) Expected (normalized) intensities of the 0- and H-beams upon stepwise shift of the third grating along the *x* direction for a monochromatic incident beam.

The results summarized in Section 3 already provide the tools required to set-up a VCN Mach-Zehnder interferometer of the triple-Laue type [3,6]. Three NP-polymer composite gratings can be adjusted in Laue geometry (transmission geometry) as shown in Figure 15a. Three gratings with equal properties can be used: each one can be tilted to a particular angle that sets its diffraction efficiency to either 0.5 or 1, so that a beam splitter (BS) or a mirror (M) is implemented for a certain incident wavelength, respectively. Varying the phase difference between the left and the right paths, one can observe intensity oscillations of the so-called 0- and H-beams [see Figure 15b] that result from the superposition of waves from the left and right beam-paths. Such a phase difference *χ* can, for instance, be obtained by stepwise rotation of a plane material slab mounted in the interferometer such that the optical path length difference between left and right paths is varied, as it is routinely done in neutron interferometry. Also, *χ* can be varied by stepwise shift of the third grating along the *x* direction. The advantage of the latter method is that the induced phase shift difference *χ* is nondispersive, *i.e.*, *χ* does not depend on the incident wavelength. Therefore, a quite broad incident wavelength spectrum—only limited by the low wavelength-selectivity of the gratings—could be used for such an instrument. Clearly, it would be more difficult to keep the third grating adjusted with respect to the first two if it must be shifted during the measurement. As an alternative that unites the advantages of both methods, certain nondispersive phase shifter configurations [79] could be used.

### 6.4. Polarizing Beam Splitters

It is an interesting question if one can produce holographic gratings whose diffraction properties can be controlled via external means—apart from tilting—such as cooling or heating, mechanical strain or electromagnetic fields. Here, the latter possibility is considered.

To account for the interaction of the neutron magnetic dipole moment $\vec{\mu }$ with the magnetic field $\vec{B}$ inside a material, the neutron-optical potential must be modified by adding a magnetic term $V_{M}=-\vec{\mu }\cdot \vec{B}\left(\vec{r}\right)$ to the nuclear term $V_{N}$. This magnetic term is related to the magnetic scattering length $b_{M}\left(\vec{Q}\right)$ that contains the magnetic form factor. The latter can be regarded as the Fourier transform of the electron-spin density of a magnetic atom. The neutron refractive index becomes

(10) $$\begin{array}{c} n=\sqrt{1-\frac{V_{N}+V_{M}}{E_{0}}}\approx 1-\lambda^{2}\frac{[b_{c}\pm b_{M}\left(0\right)]\rho }{2\pi }, \end{array}$$

where $b_{M}\left(0\right)$ refers to

(11) $$\begin{array}{c} b_{M}\left(\vec{Q}\right)=-\frac{m}{2\pi \hbar^{2}\rho }\vec{\mu }\cdot \vec{B}\left(\vec{Q}\right) \end{array}$$

at $\vec{Q}=0$. Here, *m* is neutron mass. For the holographic NP-polymer gratings, we write

(12) $$\begin{array}{c} \Delta n=\lambda^{2}\frac{[b_{c}\pm b_{M}\left(0\right)]\Delta \rho }{2\pi } \end{array}$$

for the neutron refractive index modulation amplitude in gratings with magnetic NPs. If holographic gratings contain sufficiently high concentrations of such magnetic NPs, the modulation in Equation (12) can be switched off for one spin state by adjusting the strength of an external field, *i.e.*, the grating becomes essentially transparent for neutrons in this spin state. Furthermore, by carefully choosing the grating parameters, the grating could—at the same time—act as a mirror for the other spin component. That means polarizing beam splitters for CN and VCN should be feasible.

**Figure 16:**
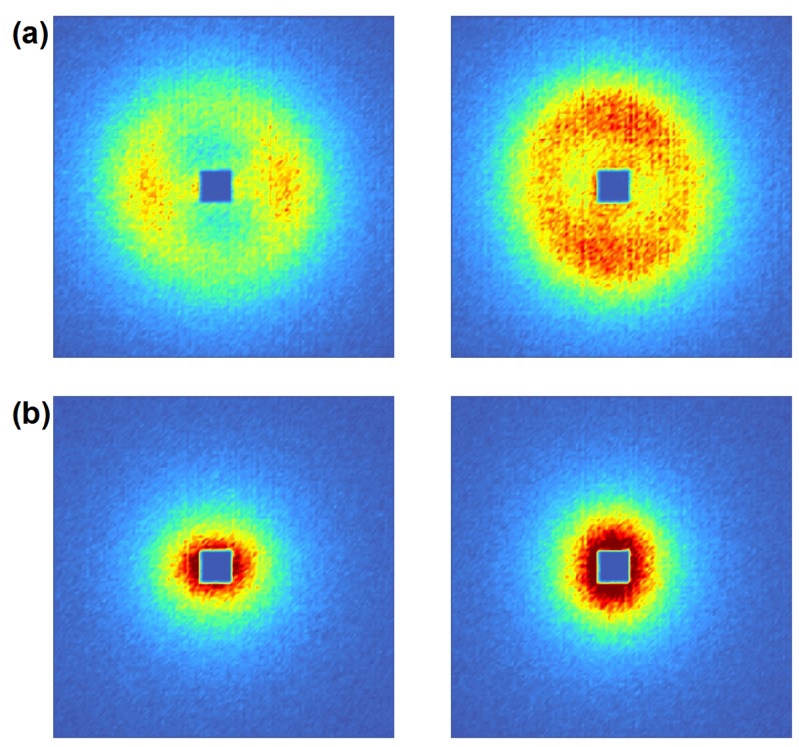
Polarized-SANS diffraction patterns of (**a**) superparamagnetic iron oxide nanocomposite (59wt% NPs) in PMMA; and (**b**) nanocomposite of ferrimagnetic CoFe_2_O_4_ (57wt% NPs) in polystyrene. The sample-detector distance is 6m and λ=0.5 nm. The external magnetic field (0.0166 T) points to the right, with spin flipper off (left) and on (right). The dark rectangular spot is the shadow of the beam stop that prevents the direct beam to reach the detector.

The superparamagnetic state of non-interacting single-domain ferro- or ferrimagnetic NPs is governed by the thermal energy and the potential energy of the particles within an external magnetic field [80]. Typically, the so-called Néel relaxation time—the time between two spin flips—is very short compared with measurement times and so the net magnetization appears to be zero without external magnetic field applied. On application of a field, the NPs get magnetized. Fortunately, compared with paramagnetism, the magnetization can become very large at comparatively low fields, depending on the type of NPs and their preparation. For instance, the maghemite NPs studied in [81] are close to saturation already at an external field of about 0.25 T. In Figure 16, data of polarized-SANS measurements at the instrument SANS I at PSI are shown. One can see the large contrast between flipper off/on (parallel—anti-parallel spin states) caused by magnetic scattering at an external field of only 0.0166 T for the superparamagnetic iron oxide NPs. From these data it also becomes clear that high concentrations of superparamagnetic nanocomposites are needed to achieve our goal. However, superparamagnetic NPs strongly absorb optical radiation. Consequently, applicability of holographic structuring is limited, especially for high NP concentration. Therefore, a two-step structuring method is being considered. In the first step, a template grating structure is recorded in H-PDLC, for instance. Second, the liquid crystals are removed from the template by a suitable solvent. The remaining voids are refilled with a solution of magnetic NPs in a photosensitive monomer. After refilling, secondary thermal- or photo-polymerization (by homogeneous illumination) ensures that the final composite grating-structure remains stable. This method is also promising for production of absorption gratings for neutrons (see Section 6.5) using Cd- or Gd-NPs.

Note that estimations of nuclear and magnetic (saturation) scattering length densities of maghemite NPs are $b_{c}\rho \approx 6.7\times 10^{18}$ *μ*m^-2^ and $b_{M}\rho \approx 1\times 10^{18}$ *μ*m^-2^, respectively (see, for instance, [82] and references therein). Materials with larger $b_{M}/b_{c}$ should be investigated in the future.

### 6.5. Absorption Gratings

**Figure 17:**
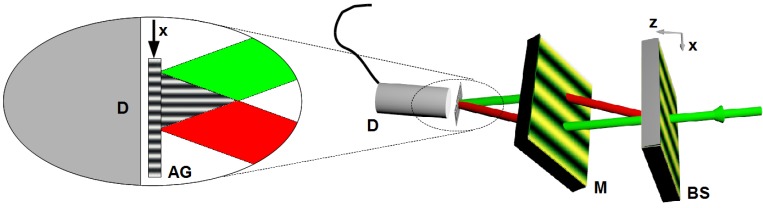
VCN Mach-Zehnder interferometer with the third grating replaced by an absorption grating (AG). The *x* position of the grating is scanned to measure the intensity modulation formed by the superposed beams with the detector (D).

The possibility to select $b_{c}$ by choosing the NP species can—apart from obtaining polarization filters or even better beam splitters and mirrors—be employed for producing absorption gratings for neutrons. Instead of SiO_2_, diamond or superparamagnetic materials one could use semiconductor CdSe NPs (quantum dots) [83,84] or Gd-containing NPs. Such gratings can serve to measure interference patterns with very small periodicity. For instance, in a Mach-Zehnder interferometer for VCN made from holographic phase gratings, a neutron interference-pattern is formed at the position of the third grating (see Figure 15). If the third grating is replaced by an absorption grating, the intensity as measured by a subsequent detector is modulated as a function of the transversal position of the absorption grating, as shown in Figure 17. Suppose that one needs a beam separation of 5 mm for inserting samples at the mirror position, for instance. Then, using a beam splitter with Λ=500 nm and incident neutron wavelength λ=4 nm, a sinusoidal interference pattern with periodicity of 500 nm is formed at a distance of 2.5 m from the beam splitter. This pattern could be observed with an absorption grating with Λ=500 nm, produced by optical holography or the two-step recording-replacement process described in Section 6.4. In Reference [85], a Zernike three-path interferometer is described. After a three-port beam splitter, two slightly slanted mirror gratings—recorded in the same sample—superpose the three coherent neutron beams at a position some meters downstream from the three-port beam splitter. In this case, a modulation of the interference pattern is observed in both longitudinal and transversal directions (*x* and *z* directions as defined in Figure 17). Upon inserting some phase-shifting material or device, the interference pattern is shifted in longitudinal direction. The pattern and its shifts can be observed using the same technique as in the Mach-Zehnder case shown in Figure 17. Another consequence of the interference of waves in thick, absorbing, periodic potentials is an effect referred to as Borrmann effect or also anomalous absorption. Its peculiarity lies in the fact that no matter how large the absorption or how thick the potential region, at least 50% of the incident radiation will be transmitted at $\theta =\theta_{B}$. One can see from dynamical diffraction theory that one of the waves interfering inside the structure has its nodes at the position of the absorbing nuclei (for crystals) or the maxima of the NP-density modulation (for NP-polymer composite gratings) and can, therefore, not be absorbed. For holographic gratings, this surprising consequence should be observable in a somewhat weakened form, because the shape of the absorbing potential is sinusoidal and is not a series of delta-peaks as it is the case for crystals. For X-rays and neutrons, observation of the effect was reported already more than five decades ago [86,87]. The phenomenon also occurs with atoms that are diffracted by standing light-waves [88]. In the latter reference, formation of standing matter-waves, as also predicted for neutrons by dynamical diffraction, is confirmed. After having observed the Pendellösung interference effect for neutron matter-waves in holographic NP-polymer gratings, it would be interesting to measure anomalous absorption in such materials.

## 7. Conclusions and Outlook

In the present paper, last decade’s development of holographic gratings for slow-neutron optics is reviewed. Production processes and recent results of CN and VCN diffraction experiments with gratings recorded in NP-polymer composites, H-PDLCs and d-PMMA are summarized.

In particular, the Pendellösung interference effect and its exploitation for reaching higher diffraction efficiency by tilting, which led to successful tests of free-standing NP-polymer composite mirrors, are explained. In Table 1, results of neutron measurements with holographic gratings are summarised.

Substitution of common hydrogen-containing monomers by deuterated material is expected to reduce the comparably high incoherent scattering/absorption of the latter materials. Such developments together with the exploration of different species of NPs is anticipated to trigger experiments such as measurement of a full Pendellösung oscillation and anomalous absorption of neutron matter-waves in holographic gratings. Also, a new kind of double-Laue grating-interferometers of the Mach–Zehnder- and Zernike-type, in which the neutron intensity oscillation is measured by scanning the transversal position of a holographic absorption grating, could become feasible. Even if usual holographic distribution of NPs is not applicable due to strong absorption of light for certain species, the removal and substitution of the liquid crystal component in H-PDLC by such NPs might be an option that is currently investigated. This method could lead to holographic polarizing beam-splitters for slow neutrons to replace costly and bulky equipment presently in use at neutron scattering facilities. Such progress will ignite rising interest in holographic gratings from the neutron scattering community, which is a prerequisite for competition and further development in our field. It should be emphasized that material science in general—but especially the field of nanomaterials—seems to continue prospering and it is expected that many more NP species, suitable for use in NP-polymer composite gratings, will be available for neutron optics applications and experiments in the future.

**Table 1:** Summarized results of neutron experiments with holographic gratings. Missing entries refer to the entry in the preceding line of the same column. The thickness is the mechanical thickness. “fsf” corresponds to free-standing film samples. “GeNF” refers to the (recently closed) Geesthacht Neutron Facility, Germany.

Sample name	Material	Λ[ nm]	Thickness [*μ*m]	*λ*[nm]	$\zeta {[}^{\circ }]$	$\eta_{P}$	Comments	Reference
G1	d-PMMA	380	$2.7\times 10^{3}$	1.5	0	0.50	D22, ILL (2003)	[66]
G2	d-PMMA	380	$2.7\times 10^{3}$	1.5	0	0.58	D22, ILL (2003)	[66]
				1		0.26		
				1.1		0.30		
				1.5		0.50		
				1.7		0.57		
				1.9		0.65		
				2.1		0.70		
				2.3		0.76		
				2.6		0.81		
				0.19		$1.3\times 10^{-3}$	S18, ILL (2009)	-
				3.8		0.75	PF2, ILL (2010)	Figure 10
						0.36	PF2, ILL (2012)	Figure 11
					40	0.30		
					50	0.27		
G3	d-PMMA	380	$3\times 10^{3}$	1.5	0	0.05	D22, ILL (2003)	[66]
10g	H-PDLC	1200	50	1.16	0	0.03	SANS-2, GeNF (2005)	[72]
				1.96		0.11		
10f	H-PDLC	1200	50	1.16	0	0.03	SANS-2, GeNF (2005)	[89]
				1.96		0.11		-
				4.4		0.31	PF2, ILL (2012)	Figure 12
29w	H-PDLC	560	50	1.16	0	0.009	SANS-2, GeNF (2008)	[32]
29e	H-PDLC	1000	100	1.16	0	0.005	SANS-2, GeNF (2008)	-
S#2 (NP2007)	20 vol% SiO_2_ NP	1000	50	0.19	0	$4\times 10^{-4}$	S18, ILL (2009)	[31]
				1.7	0	0.07	SANS I, PSI (2010)	[46]
					45	0.11		-
					58	0.20		[46]
S#4 (NP2007)	20 vol% ZrO_2_ NP	1000	48	0.19	0	$1.5\times 10^{-4}$	S18, ILL (2009)	-
				1.16	0	0.01	SANS I, PSI (2010)	[59]
S#1 (NP2010b)	34 vol% SiO_2_ NP	1000	46	1.7	0	0.08	SANS I, PSI (2010)	[46]
S#3 (NP2010b)	34 vol% SiO_2_ NP	500	53	1.7	0	0.03	SANS I, PSI (2010)	-
S#5 (NP2010b)	20 vol% SiO_2_ NP	1000	96	1.7	0	0.19	SANS I, PSI (2010)	[46]
					56	0.29	-”-, 3-port beam splitter	[85]
S#7 (NP2010b)	20 vol% SiO_2_ NP	500	101	1.7	0	0.06	SANS I, PSI (2010)	[46]
					66	0.11		-
				2	64	0.50	-”-, beam splitter	[46,59]
				3.8	0	0.25	PF2, ILL (2010)	[46]
				3.8	43	0.31		
				3.8	60	0.61		
				3.8	65	0.83		
S#9 (NP2010b)	20 vol% SiO_2_ NP	1000	186	3.8	0	0.50	PF2, ILL (2012), strong $\eta_{\pm 2}$	-
				3.8	45	0.52		Figure 4
				3.8	60	0.35		-
S#11 (NP2010b)	20 vol% SiO_2_ NP	500	180	1.7	0	0.04	SANS I, PSI (2010)	[46]
S#1 (NP2011b)	20 vol% SiO_2_ NP	500	86	4.5	0	0.20	PF2, ILL (2011)	Figure 5
S#2 (NP2011b)	25 vol% SiO_2_ NP	500	71	4.5	0	0.20	PF2, ILL (2011)	Figure 5
S#10 (NP2011b)	20 vol% SiO_2_ NP	500	136	4.5	0	0.08	PF2, ILL (2011), fsf	-
S#11 (NP2011b)	25 vol% SiO_2_ NP	500	115	4.4	0	0.12	PF2, ILL (2011), fsf	-
S#6 (NP2011b)	20 vol% SiO_2_ NP	500	96	4.4	0	0.20	PF2, ILL (2011)	Figure 7
						0.20	-”-, fsf	
S#7 (NP2011b)	20 vol% SiO_2_ NP	500	115	4.4	0	0.25	PF2, ILL (2011), fsf	-
				4.1	70	0.90	-”-, mirror	[60]
